# Peptide inhibitors of dengue virus and West Nile virus infectivity

**DOI:** 10.1186/1743-422X-2-49

**Published:** 2005-06-01

**Authors:** Yancey M Hrobowski, Robert F Garry, Scott F Michael

**Affiliations:** 1 Department of Microbiology and Immunology, Tulane University Health Sciences Center, New Orleans, Louisiana 70112 USA; 2 Graduate Program in Cellular and Molecular Biology, Tulane University, New Orleans, LA 70112 USA; 3 Biotechnology Program, Florida Gulf Coast University, Fort Myers, FL 33965 USA

## Abstract

Viral fusion proteins mediate cell entry by undergoing a series of conformational changes that result in virion-target cell membrane fusion. Class I viral fusion proteins, such as those encoded by influenza virus and human immunodeficiency virus (HIV), contain two prominent alpha helices. Peptides that mimic portions of these alpha helices inhibit structural rearrangements of the fusion proteins and prevent viral infection. The envelope glycoprotein (E) of flaviviruses, such as West Nile virus (WNV) and dengue virus (DENV), are class II viral fusion proteins comprised predominantly of beta sheets. We used a physio-chemical algorithm, the Wimley-White interfacial hydrophobicity scale (WWIHS) [1] in combination with known structural data to identify potential peptide inhibitors of WNV and DENV infectivity that target the viral E protein. Viral inhibition assays confirm that several of these peptides specifically interfere with target virus entry with 50% inhibitory concentration (IC50) in the 10 μM range. Inhibitory peptides similar in sequence to domains with a significant WWIHS scores, including domain II (IIb), and the stem domain, were detected. DN59, a peptide corresponding to the stem domain of DENV, inhibited infection by DENV (>99% inhibition of plaque formation at a concentrations of <25 μM) and cross-inhibition of WNV fusion/infectivity (>99% inhibition at <25 μM) was also demonstrated with DN59. However, a potent WNV inhibitory peptide, WN83, which corresponds to WNV E domain IIb, did not inhibit infectivity by DENV. Additional results suggest that these inhibitory peptides are noncytotoxic and act in a sequence specific manner. The inhibitory peptides identified here can serve as lead compounds for the development of peptide drugs for flavivirus infection.

## Introduction

Enveloped viruses utilize membrane-bound fusion proteins to mediate attachment and entry into specific target host cells. During the virion assembly process, newly synthesized envelope proteins are targeted to the endoplasmic reticulum and golgi apparatus where initial folding and post-transcriptional processing occurs, including multimerization, glycosylation, and proteolysis. This initial folding and processing is required to achieve a conformation where the proteins are held in a metastable state prior to virion release. Post virion release, the multimeric envelope proteins are poised to undergo structural rearrangement leading to fusion of the virion and the new target cell lipid bilayer membranes. Depending on the virus system, the rearrangement trigger can take the form of specific receptor binding, multiple receptor binding, decreased pH following receptor mediated endocytosis, or a combination of triggers.

The prototypic viral envelope fusion protein, the hemagglutinin of influenza virus, contains short alpha helical domains in the trimeric virion configuration. In response to receptor binding and decreased pH, the short helices rearrange with adjoining sequences to produce a longer helix, thus exposing an N-terminal fusion peptide that is believed to interact directly with the target cell membrane. This is followed by a hinge-like bending of the entire complex to adjoin and fuse the two lipid membranes [2,3]. The structural rearrangements that result in extrusion of the fusion peptide and subsequent collapse involve alterations in packing between regions both within individual fusion proteins as well as between monomeric subunits in the trimeric structures. Several disparate viruses, including arenaviruses, coronaviruses, filoviruses, orthomyxoviruses, paramyxoviruses and retroviruses, encode similar proteins that together are classified as class I fusion proteins. These class I viral fusion proteins vary in length and sequence, but are similar in overall structure [4,5].

Qureshi et al. (1990) demonstrated that a peptide from one of the two extended helical domains of the HIV-1 transmembrane protein can block virion infectivity. Subsequently, the FDA approved anti-HIV-1 drug Fuzeon™ (aka DP178, T-20, enfuvirtide) and other N- and C-helix inhibitory peptides were developed [6,7]. These results have greatly motivated the search for other HIV-1 inhibitory peptides [8,9]. Additional peptide mimics of the fusion proteins of other retroviruses, and of orthomyxoviruses, paramyxoviruses, filoviruses, coronaviruses, and herpesviruses have also been identified and shown to inhibit viral entry [10-18]

The envelope fusion proteins of several virus types, including the flaviviruses and alphaviruses, have a structure distinct from class I viral fusion proteins. The envelope glycoprotein (E) of the flavivirus tick-borne encephalitis virus (TBEV) consists of three domains: a structurally central amino terminal domain (domain I), a dimerization domain (domain II) and a carboxyl terminal Ig-like domain (domain III), all containing predominantly beta sheet folds [19]. The primary sequence of E1, the fusion protein of Semliki Forest virus, an alphavirus, revealed a remarkable fit to the scaffold of TBEV E [20] suggesting the existence of a second class of viral fusion proteins. The dengue virus (DENV) E protein has also been shown to have a class II structure [21]. Recent studies of flavivirus virions and proteins by cryoelectron microscopy and crystal structure analysis have lead to a greatly increased understanding of the function of these class II viral envelope proteins, including the structural rearrangements they undergo during maturation, triggering and fusion [21-28].

The flaviviruses, which include DENV, West Nile virus (WNV), yellow fever virus, Japanese encephalitis virus (JEV), and TBEV, among others, are transmitted between vertebrate hosts by insect vectors. The most serious manifestations of DENV infection are dengue hemorrhagic fever (DHF) and dengue shock syndrome (DSS). There are four serotypes of DENV (1–4), which together cause an estimated 50 million human infections per year [29], and each can cause DF, DHF or DSS. Because of the phenomenon of antibody-dependent enhancement (ADE), or other immune phenomena, protection against one DENV serotype increases the risk of DHF or DSS when the individual is exposed to another serotype [30-32]. Cross-reactive, but non-neutralizing antibodies can mediate entry of DENV into macrophages, dendritic cells and other viral target cells via Fc receptors, increasing virus titers and thus pathology. Multivalent DENV vaccines have shown some promise in humans [32-39] and in nonhuman primate studies [40,41], but face several obstacles. Antiviral drugs, which target each of the four serotypes of DENV without enhancing pathogenesis of any serotype, are urgently needed. The recent introduction of WNV in the United States further highlights the public health challenges posed by flaviviruses. No effective vaccine or antiviral drug therapy is currently available against either DENV or WNV.

Although there are many differences between the structures of class I and class II viral fusion proteins, we hypothesized that they function through a similar membrane fusion mechanism involving rearrangements of domains, and that peptides mimicking portions of class II viral fusion proteins would inhibit virion fusion and entry steps thereby serving as lead compounds for the development of antivirals. We used a physio-chemical algorithm, the Wimley-White interfacial hydrophobicity scale [1] in combination with known structural data to predict regions of the DENV and WNV E proteins that may play roles in protein-protein rearrangements or bilayer membrane interactions during the entry and fusion process. Several of these peptides specifically inhibit DENV or WNV infection.

## Results

### Identification of Flavivirus inhibitory peptides

**Figure 1:**
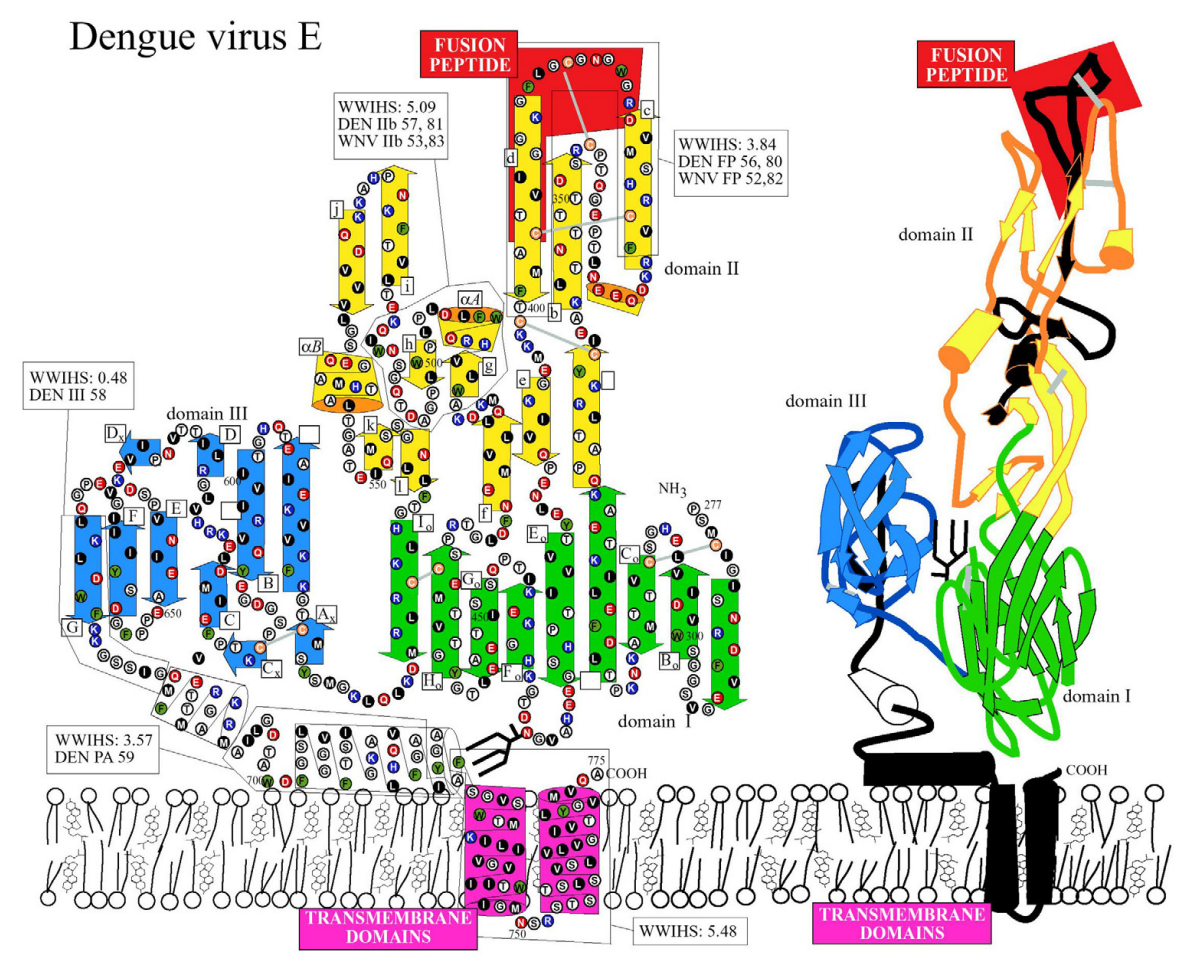
**Diagramatic structure of DENV envelope protein showing inhibitory peptide regions**. Grey lines: dicysteine linkages. Black stick figures: N-glycosylation sites. Regions with significant Wimley-White interfacial hydrophobicity scale scores were predicted with MPeX (Boxed in left depiction; black in right depiction). Sequences of DENV peptides and the location of WNV homologs are indicated.

The domains that precede the transmembrane anchors of most class I fusion proteins are not highly hydrophobic, however, they usually contain a cluster of aromatic amino acids and display a tendency to partition into bilayer membranes, as revealed by analyses using the experimentally-determined Wimley-White interfacial hydrophobicity scale (WWIHS) [42]. Fuzeon's corresponding sequence overlaps the aromatic pre-anchor domain of HIV-1 TM. Synthetic peptides corresponding to other domains of class I viral fusion proteins with significant WWIHS scores may also inhibit viral infectivity [43]. Previously, we suggested that peptide drugs analogous to Fuzeon might be developed for HCV and other members of the Flaviviridae [44]. DENV E contains five domains with significant WWIHS scores (Fig. 1, WWIHS sequences in black). These include the fusion peptide domain, a portion of subdomain IIb, the pre-anchor stem region following domain III, and the transmembrane domain. Sequences with high WWIHS scores are similarly located in the X-ray structures of WNV E and alphavirus (SFV and Sindbis virus – SINV) E1, and potentially also in the putative class II fusion proteins of hepatitis C virus (HCV), pestiviruses and bunyaviruses [44,45]. Regions with high WWIHS scores are predicted to play a role in protein-protein interactions during structural rearrangements or protein-lipid interactions during bilayer fusion, and we predicted that synthetic peptides corresponding to these regions may have the potential to inhibit flavivirus infectivity.

**Figure 2:**
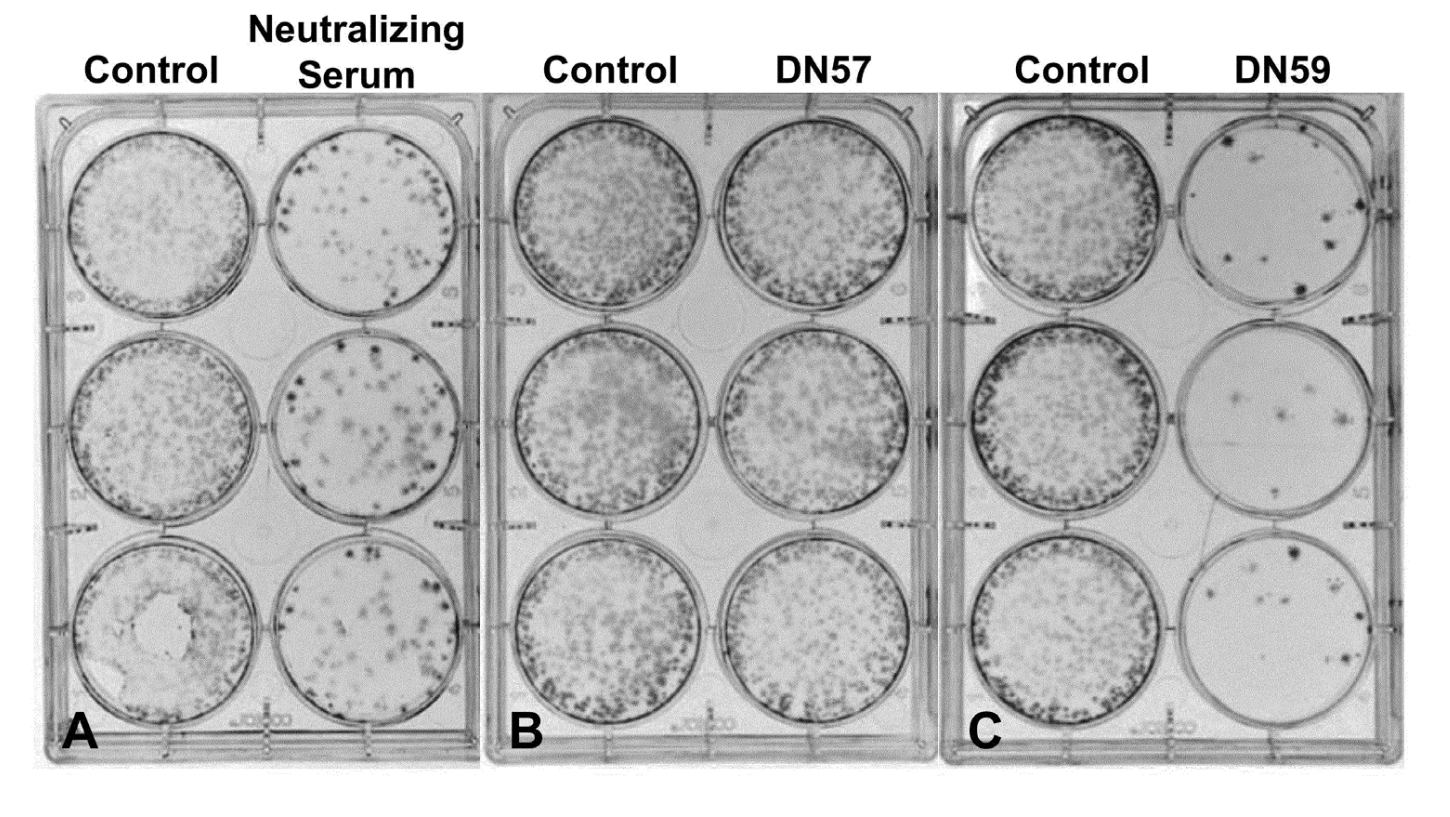
**Plaque inhibition assay**. (A) Preincubation of DENV with neutralizing antiserum reduces plaque number by 74%. (B) Preincubation of DENV with a non-inhibitory peptide shows no reduction in plaques. (C) Preincubation of DENV with one of the inhibitory peptides (DN59) shows a greater than 95% reduction in plaques.

To test this hypothesis, an initial set of synthetic peptides representing sequences of DENV E and WNV E with significant WWIHS scores was synthesized and screened for the ability to inhibit plaque formation by these flaviviruses (Table 1). Peptides corresponding to the transmembrane domain were not tested because this region is not exposed during the entry process. Initial assays for inhibitory activity were performed using the highest concentration of each peptide that could be obtained in aqueous solution with a maximum of 1% DMSO (between 29 and 128 μM). Plaque reduction in which the inhibitor is removed after virus adsorption is the most stringent test of an antiviral agent. Prior to initiating these studies, we developed a new immunoplaque assay for DENV and WNV. Approximately 200 focus forming units (FFU) of either WNV or DENV were preincubated with each of the peptides and used to infect monolayers of LLCKM-2 monkey kidney epithelial cells. The number of resulting viral foci was determined from three experiments and normalized to a no-peptide control to calculate the percent inhibition. Our screening of this initial set detected several peptides that were able to inhibit infection by DENV or WNV (Table 1, Fig. 2). Peptides similar in sequence to domains with a significant WWIHS scores, including domain II (IIb) (WN53 and WN83), and stem domain (DN59), were found to have inhibitory activity.

**Table 1:** Initial peptides synthesized and tested for inhibitory activity.

**Peptide**	**Sequence**	**Location**^a^	**Concentration (μM)**	**% Inhibition +/- SD**
DN80	MVDRGWGNHAGLFGKGSIV	386–400	49.9	17 +/- 10
DN57	AWLVHTQWFLDLPLPWLPGADTQGSNWI	485–503	30.6	7 +/- 4
DN81	AWLVHRQWFLDLPLPWLPG	485–512	42.6	25 +/- 8
DN59	MAILGDTAWDFGSLGGVFTSIGKALHQVFGAIY	692–724	29.0	93 +/- 2
WN82	VVDRGWGNGAGLFGKGSID	396–410	52.5	4 +/- 13
WN53	TFLVHREWFMDLNLPWSSAGSTVWR	500–518	98.7	56 +/- 5
WN83	TFLVHREWFMDLNLPWSSA	500–524	128.0	70 +/- 2

^a ^numbering from the beginning of the E polyprotein in either DENV or WNV.

### Determination of 50% inhibitory concentrations

**Figure 3:**
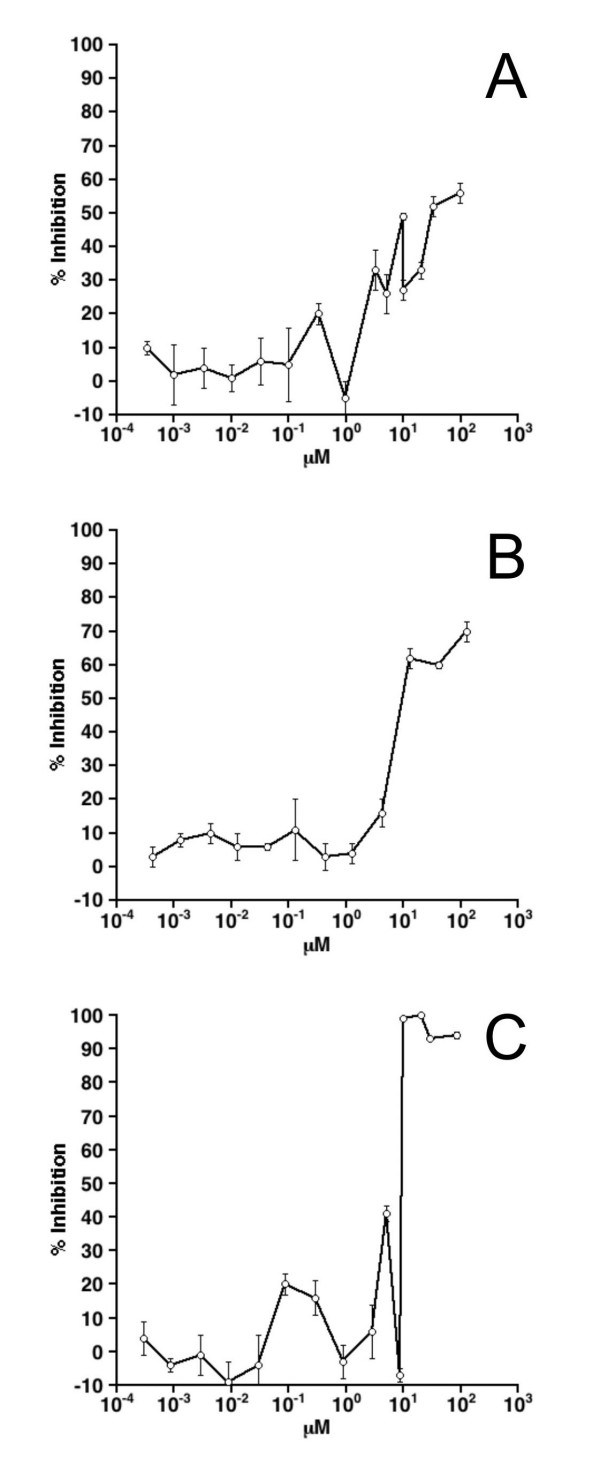
**Dose-response curves for WN53, WN83 and DN59 peptides**. (A) Increasing concentrations of peptide WN53 produce a corresponding increase in WNV inhibition with an IC50 in the 10μ range. (B) Increasing concentrations of peptide WN83 produce a corresponding increase in WNV inhibition with an IC50 in the 10 μM range. (C) Increasing concentrations of peptide DN59 also produce a corresponding increase in DNV inhibition with an IC50 in the 10 μM range. All measurements were made in triplicate, with mean +/- SD shown.

Dose-response curves were determined for the most potent of the peptides WN53 and WN83 against WNV and also for peptide DN59 against DENV (Fig. 3). The WN53 peptide showed a maximum inhibitory activity against WNV of 56.0 +/- 3.0% (mean +/- SD) at 99 μM. The inhibitory activity decreased with decreasing concentration with a 50% inhibitory concentration (IC50) at roughly 10 μM. The WN83 peptide showed a maximum inhibitory activity against WNV of 70.0 +/- 3.0% at 128 μM. The inhibitory activity decreased with decreasing concentration with an IC50 of roughly 10 μM. The DN59 peptide showed a maximum inhibitory activity against DENV of 100.0 +/- 0.5% at 20 μM. The inhibitory activity decreased with decreasing concentration with an IC50 of at roughly 10 μM. DENV stem peptide 59 (DN59) and WNV peptides 53 and 83 (WN53, WN83) reproducibly inhibited infectivity at low μM concentrations.

### Specificity of peptide inhibitory activity

**Figure 6:**
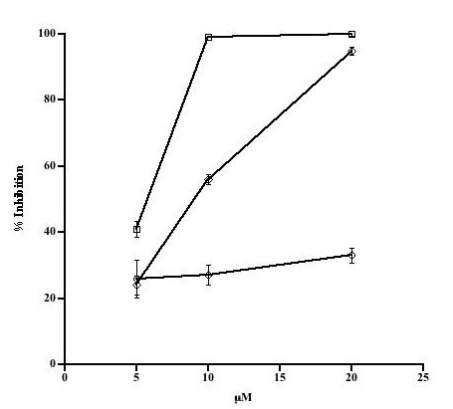
**Inhibitory effect of peptides WN53 and DN59 alone and in combination**. WN53 and DN59 peptides were tested alone (■ DN59 alone, ● WN53 alone) or together (◆) with WNV and inhibitory activity at three concentrations was measured (mean of three trials +/- SD shown). DN59 and WN53 together have an effect intermediate between the two peptides alone.

The DN59 peptide matches a pre-anchor domain sequence that is highly conserved among insect-transmitted flaviviruses. DN59 inhibited infection by DENV (>99% inhibition of plaque formation at a concentrations of <25 μM). Cross-inhibition of WNV fusion/infectivity (>99% inhibition at <25 μM) was also reproducibly demonstrated with DN59 (Fig. 6). However, WNV inhibitory peptide WN83 did not inhibit infectivity by DENV.

**Figure 4:**
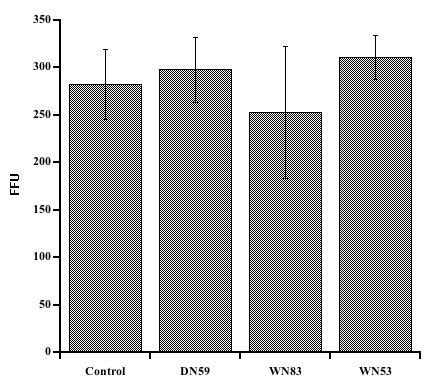
**Effect of DENV and WNV specific peptides against another virus**. Peptides DN59, WN83 and WN53 at 100 μg/ml concentrations were tested for inhibitory activity against the alphavirus SINV in a similar plaque reduction assay. Results are shown as the mean of three trials +/- SEM. None of the peptides showed a statistically significant inhibitory effect against SINV (ANOVA, p = 0.705, with Dunnett's posthoc test).

**Figure 5:**
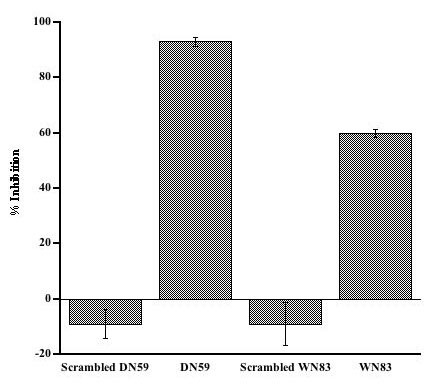
**Effect of scrambled peptide order on inhibitory function**. Scrambled versions of peptides DN59 and WN83 at 100 μg/ml concentrations were tested for inhibitory effect against DENV and WNV, respectively. Scrambled versions of the peptides showed no inhibitory activity compared to the original DN59 and WN83 peptides.

To determine if these peptides specifically inhibit infectivity of the viruses for which they were designed, the WN53, WN83 and DN59 peptides were tested for inhibitory effects against Sindbis virus (SINV), an alphavirus that encodes a class II fusion protein. None of the peptides showed a statistically significant effect against SINV infectivity (Fig. 4). Peptides with the same amino acid composition as WN83 and DN59, but a scrambled sequence (Scrambled WN83: VATWHLDWSREFPLFLMNS; Scrambled DN59: YFIDTSGAIWGASHLTGVLFDFMGIQGGAVLAK) were synthesized and tested for the ability to inhibit infection by WNV and DENV respectively. Neither scrambled peptide significantly inhibited infection by these viruses (Fig. 5). These results provide evidence that the action of these inhibitory peptides not due to general inactivation of enveloped virions and is sequence specific.

### Peptide toxicity

**Figure 7:**
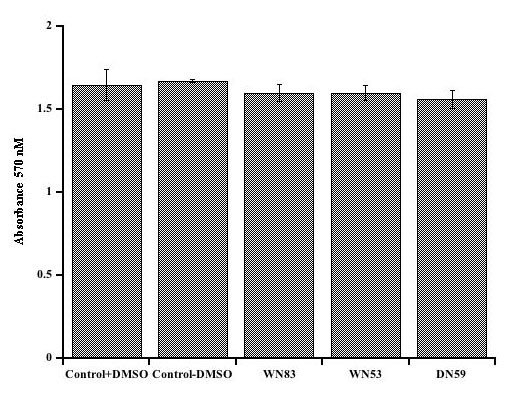
**Toxicity of inhibitory peptides in cell culture**. MTT assays for cell viability were performed after 24 hr incubation of cells with 100 μg/ml of peptides WN83, WN53, and DN59 (mean from three experiments +/- SEM). No statistically significant differences in cell viability were observed (ANOVA p = 0.672, with Dunnett's posthoc test).

It is possible that inhibitory peptides induce cellular alterations or toxicity that can block flavivirus entry or other steps in the replication cycle. To address this possibility, LLCMK-2 monkey kidney epithelial cell monolayers were exposed to 100 mg/ml concentrations of WN53, WN83 and DN59 peptides for 24 hrs, and cell viability was assayed with an MTT assay. No statistical difference was observed between the viability of control cells versus cells exposed to the peptides or DMSO (Fig. 7). This result suggests that these inhibitory peptides are not blocking infectivity via effects on host cell metabolism or viability.

### Non-synergistic activity of combined peptides

When added in combination, peptides that block entry at different steps or that target different domains may produce greater inhibition of DENV-2 infectivity than either peptide alone. Synergistic or antagonistic effects are also possible, if a peptide that alters protein-protein interactions allows greater or lesser access to E domains targeted by another peptide. Since the WN53, WN83 and DN59 peptides all inhibited WNV entry, the possibility of antagonistic or synergistic function was examined by testing WN53 and DN59 alone or in combination at three concentrations (5, 10 and 20 μM). At all three concentrations, the peptide combination was more effective than WN53 alone, but less effective than DN59 alone. This indicates that the activity of the WN53 peptide has an antagonistic effect on the function of the DN59 peptide (Fig. 6).

## Discussion

Synthetic peptides corresponding to sequences in DENV and WNV E proteins were identified that inhibited infectivity of these viral pathogens of major public health importance. The inhibitory effects of these peptides were dose dependent with IC50s in the range of 10 μM. Several of the most potent of these peptides showed no inhibitory activity against SINV, an alphavirus that possesses a class II viral fusion protein with a similar overall structure as flavivirus E. Scrambled peptides with the same amino acid composition as the inhibitory peptides, but with a different primary sequence, failed to inhibit DENV and WNV infection. None of the DENV or WNV inhibitory peptides induced gross cytopathic effects, killing of cultured cells or showed evidence of *in vitro *cellular toxicity. These results indicate that these inhibitory peptides function through a sequence-specific mechanism and are not merely cytotoxic.

Membrane fusion by both class I and II viral fusion proteins is initiated by interaction of the fusion peptide with the target cell membrane. In class I viral fusion proteins, a subsequent rearrangement of a trimer of the proteins, each with two α helices, to form a six-helix bundle brings the viral and cell membranes into closer proximity. Inhibitors of viruses with class I fusion proteins, such as Fuzeon™ that mimic a portion of one or the other of the two α helices, interfere with a step proximal to six-helix bundle formation possibly by forming an inactive aggregate with the opposite helix. Recent studies indicate that after insertion of the fusion peptide, class II viral fusion proteins likewise undergo rearrangements. In this case, intraprotein interactions may occur between the stem domain and domains I, II and/or III [21-28,46]. According to this model, the viral and cellular membranes are brought closer by interactions of the stem with other portions of E, resulting in bilayer fusion. DN59, WN53 and WN83 peptides may interfere with the intramolecular interactions between the stem and other portions of class II viral fusion proteins, a possibility suggested previously [23,24,46].

Two of the inhibitory peptides (WN53 and WN83) are designed from overlapping regions of the E protein domain I/II junction and are specifically inhibitory against WNV. Recently, other investigators have hypothesized that small molecule inhibitors to this domain I/II junction region might be developed. Modis et al (2003; 2004) predicted that interactions near this region (the k-l loop) that are involved in the rotational changes between these domains might be blocked by small molecule inhibitors. However, our similar peptides designed from the analogous region of the DENV E protein (D57 and D81) failed to inhibit DENV infectivity.

The possibility that WN53, WN83 and DN59 interact with some target cell surface component to exert their inhibitory effects cannot be ruled out. However, the majority of flavivirus neutralizing antibodies that appear to be involved in receptor blocking bind to domain III, and soluble domain III itself can block flavivirus entry, apparently through competition for cellular receptors [47-51]. In contrast, the domains that correspond to WN53, WN83 and DN59 peptides, IIb and the pre-anchor stem, appear to be involved in structural rearrangements during fusion, rather than direct interactions with cellular receptors. Interestingly, previous observations indicate that some monoclonal antibodies block virion entry at a post attachment step, indicating that they may interfere with conformational changes necessary for fusion [52]. That possibility that some antibodies can gain access to regions important for conformational changes and block these changes suggests that these inhibitory peptide regions might be candidates for novel vaccine designs that either utilize the inhibitory peptide regions directly as antigens, or target the regions that interact with the inhibitory peptides. Further studies are needed to define the exact mechanism of inhibition of these DENV and WNV peptides, and the specific nature or location of their interactions with viral targets.

The DN59 peptide is inhibitory against DENV as well as WNV. The corresponding pre-anchor region is highly conserved between DENV and WNV as well as among other flaviviruses (Table 2) and probably functions in a similar manner during entry of all flaviviruses [53]. Thus, DN59 or similar peptides may act as broad-spectrum flavivirus inhibitors. Other flaviviruses considered potential bioterrorism agents, including JEV, Kyasanur Forest disease virus and TBEV, may also be inhibited by DN59, a DN59 derivative, or by an analogous peptide. Unlike proposed DENV vaccines, which must be multivalent (ie. simultaneously effective against each of the four DENV serotypes because of the phenomenon of antibody dependent enhancement), peptide drugs targeting the highly conserved stem conserved motifs in flavivirus E may demonstrate cross-strain efficacy.

**Table 2:** Alignment of preanchor domain sequences from representative flaviviruses.

*Virus*^*a*^	*Pre-anchor sequence*^*b*^	*Location*^*c*^
DENV-1	AILGDTAWDFGSIGGVFTSVGKLIHQIFGTA	693–723
DENV-2	AILGDTAWDFGSLGGVFTSIGKALHQVFGAI	693–723
DENV-3	AILGDTAWDFGSVGGVLNSLGKMVHQIFGSA	691–721
DENV-4	AILGETAWDFGSVGGVLTSLGKAVHQVFGSV	692–722
WNV	AVLGDTAWDFGSVGGVFTSVGKAVHQVFGGA	709–739
YFV	AVMGDTAWDFSSAGGFFTSVGKGIHTVFGSA	695–725
SLEV	AVLGDTAWDFGSIGGVFTSIGKALHQVFGGA	707–737
JEV	AALGDTAWDFGSIGGVFNSIGKAVHQVFGGA	712–742
TBEV	TVIGEHAWDFGSAGGFLSSIGKAVHTVLGGA	694–724
OHFV	TVLGEHAWDFGSTGGFLSSIGKALHTVLGGA	694–724
KFV	TVVGEHAWDFGSVGGMLSSVGKALHTAFGAA	695–725
POWV	SVVGEHAWDFGSVGGVLSSVGKAIHTVLGGA	693–723

^a^DENV: dengue virus; WNV: West Nile virus; YFV: yellow fever virus; SLEV: St. Louis encephalitis virus; JEV: Japanese encephalitis virus; TBEV: Tick-borne encephalitis virus; OHFV: Omsk hemorrhagic fever virus; KFV: Kyasanur Forest virus; POWV: Powassan virus

^b ^* = identical or chemically similar amino acids in every sequence

^c^numbering from the beginning of the E polyprotein.

IC50s in the μM range have been considered promising for class I viral fusion protein inhibitor development [54,55]. Thus, the peptides identified here can serve as lead compounds that may be developed as peptide drugs against the four serotypes of DENV, WNV and potentially other flaviviruses. We anticipate that such peptide inhibitors may be as successful as the HIV-1 inhibitory peptide Fuzeon™. Unlike persistant HIV infections, immune responses against DENV and other flaviviruses are capable of clearing the viruses in individuals that survive the initial infection. By reducing the viral load during the initial stages of infection, it may be possible to extend the window of time during which an immune response could arise, and thus enable more individuals to control, eliminate and survive infections by these agents. Evidence for the ability to therapeutically intervene in flavivirus-induced diseases has been demonstrated with the recent observation that administration of neutralizing antibodies against WNV can be curative, even after symptom initiation [56]. Development of resistant mutants will be a concern, but should be a less problematic than in the case of long-term treatment of persistent retroviral infections.

It is worth noting that the HIV inhibitor Fuzeon™, was initially identified using a predictive strategy without the availability of structural data [6,7,57]. The fact that we developed these peptides using a predictive algorithm validates our approach as well as the accuracy of the flavivirus E protein structural data. A similar approach may be useful for the large number of other viruses with class II envelope fusion proteins with or without known structures.

## Materials and methods

### Design and synthesis of peptides

Sequences of DENV and WNV E with positive Wimley-White interfacial hydrophobicity scale scores were determined using the program Membrane Protein eXplorer [1]. After consideration of the known secondary structures for several subdomains of E, selected peptides were synthesized by solid-phase conventional N-α-9-flurenylmethyloxycarbonyl chemistry (Genemed Synthesis, San Francisco, CA). Peptides were purified by reverse-phase high performance liquid chromatography and confirmed by amino acid analysis and electrospray mass spectrometry. Peptide stock solutions were prepared in 20% (v/v) dimethyl sulfoxide (DMSO): 80% (v/v) H_2_0, and concentrations determined by absorbance of aromatic side chains at 280 nm. Scrambled peptides sequences were obtained by drawing from a hat.

### Viruses and Cells

DENV strain New Guinea-2 and WNV strain Egypt 101 were obtained from R. Tesh at the World Health Organization Arbovirus Reference Laboratory at the University of Texas at Galveston. DENV and WNV were propagated in the African green monkey kidney epithelial cell line, LLCKM-2, a gift of K. Olsen at Colorado State University. Sindbis virus (SINV) containing the enhanced green fluorescent protein (EGFP) protein expression cassette was obtained from K. Ryman at Louisiana State University at Shreveport and was propagated in baby hamster kidney cells. All cells were grown in Dulbecco's modified eagle medium (DMEM) with 10% (v/v) fetal bovine serum (FBS), 100 U/ml penicillin G and 100 mg/ml streptomycin, at 37°C with 5% (v/v) CO_2_.

### Viral plaque reduction assays

LLCKM-2 target cells were seeded at a density of 3 × 10^5 ^cells in each well of a 6-well plate 48 h prior to infection. Approximately 200-focus forming units (FFU) of DENV, WNV, or SINV/EGFP were incubated with or without peptides in serum-free DMEM for 1 h at rt. Virus/peptide or virus/control mixtures were allowed to infect confluent LLCKM-2 monolayers for 1 h at 37°C, after which time the medium was removed and the cells were washed once with phosphate buffered saline (PBS) and overlaid with fresh DMEM/10% (v/v) FBS containing 0.85% (w/v) SeaPlaque Agarose (Cambrex Bio Science, Rockland, ME). Cells were then incubated at 37°C with 5% CO_2 _for 1 day (Sindbis virus), 3 days (WNV) or 6 days (DNV). Sindbis virus infections were quantified by directly counting green fluorescing foci. Cultures infected with DENV were fixed with 10% formalin overnight at 4°C and permeablized with 70% (v/v) ethanol prior to immunostaining and visualization using a human polyclonal anti-flavivirus antibody (a gift of V. Vorndam, CDC, San Juan) followed by horseradish peroxidase (HRP) conjugated goat anti-human immunoglobulin (Pierce, Rockford, IL) and AEC chromogen substrate (Dako, Carpinteria, CA). WNV plaques were similarly visualized using a mouse anti-WNV antibody (Chemicon, Temecula, CA) and an HRP conjugated goat anti-mouse antibody (Dako, Carpinteria, CA).

### Toxicity assay

Peptide cytotoxicity was measured using the TACS™ MTT cell proliferation assay (R&D systems, Minneapolis, MN) according to the manufacturer's instructions.
